# Spatial variation and temporal trends of testicular cancer in Great Britain

**DOI:** 10.1054/bjoc.2001.1739

**Published:** 2001-06

**Authors:** M B Toledano, L Jarup, N Best, J Wakefield, P Elliott

**Affiliations:** The Small Area Health Statistics Unit, Department of Epidemiology and Public Health, Imperial College School of Medicine, Norfolk Place, London W2 1PG, UK

**Keywords:** testicular cancer, temporal trends, geographical, epidemiology

## Abstract

Increases in testicular cancer incidence have been reported in several countries over a long period. Geographical variability has also been reported in some studies. We have investigated temporal trends and spatial variation of testicular cancer at ages 20–49 in Britain. Temporal trends in testicular cancer incidence were examined, 1974 to 1991 and in mortality, 1981–1997. Spatial variation in incidence was analysed across electoral wards, 1975 to 1991. We used Poisson regression to examine for regional and socio-economic effects and Bayesian mapping techniques to analyse small-area spatial variability. Incidence increased from 6.5 to 11.1 per 100 000 in men at ages 20–34, and from 5.6 to 9.7 per 100 000 in men at ages 35–49, while mortality declined by 50% in both age groups. Risks of testicular cancer varied across regional cancer registries, ranging from 0.79 (95% CI: 0.73–0.84) to 1.32 (95% CI: 1.25–1.38), and was higher in the most affluent compared with the most deprived areas. Analyses within 2 regions (one predominantly urban, the other predominantly rural) did not indicate any localized geographical clustering. The increasing incidence contrasted with a decreasing mortality over time in Great Britain, similar to that found in other countries. The higher risk in more affluent areas is not consistent with findings on social class at the individual level. The absence of any marked geographical variability at small area scale argues against a geographically varying environmental factor operating strongly in the aetiology of testicular cancer. © 2001 Cancer Research Campaign http://www.bjcancer.com


British Journal of Cancer (2001) 84(11), 1482–1487
© 2001 Cancer Research Campaign
doi: 10.1054/ bjoc.2001.1739, available online at http://www.idealibrary.com on IDEAL®

http://www.bjcancer.com

### Spatial variation and temporal trends of testicular cancer in Great Britain

**MB Toledano, L Jarup, N Best, J Wakefield and P Elliott**

The Small Area Health Statistics Unit, Department of Epidemiology and Public Health, Imperial College School of Medicine, Norfolk Place, London W2 1PG, UK

**Summary** Increases in testicular cancer incidence have been reported in several countries over a long period. Geographical variability has also been reported in some studies. We have investigated temporal trends and spatial variation of testicular cancer at ages 20–49 in Britain. Temporal trends in testicular cancer incidence were examined, 1974 to 1991 and in mortality, 1981–1997. Spatial variation in incidence was analysed across electoral wards, 1975 to 1991. We used Poisson regression to examine for regional and socio-economic effects and Bayesian mapping techniques to analyse small-area spatial variability. Incidence increased from 6.5 to 11.1 per 100 000 in men at ages 20–34, and from 5.6 to 9.7 per 100 000 in men at ages 35–49, while mortality declined by 50% in both age groups. Risks of testicular cancer varied across regional cancer registries, ranging from 0.79 (95% CI: 0.73–0.84) to 1.32 (95% CI: 1.25–1.38), and was higher in the most affluent compared with the most deprived areas. Analyses within 2 regions (one predominantly urban, the other predominantly rural) did not indicate any localized geographical clustering. The increasing incidence contrasted with a decreasing mortality over time in Great Britain, similar to that found in other countries. The higher risk in more affluent areas is not consistent with findings on social class at the individual level. The absence of any marked geographical variability at small area scale argues against a geographically varying environmental factor operating strongly in the aetiology of testicular cancer. © 2001 Cancer Research Campaign http://www.bjcancer.com

**Keywords**: testicular cancer; temporal trends; geographical; epidemiology

Cancer of the testis is a malignant tumour, which mainly affects young men, with a peak incidence at around 30 years of age (Senturia, 1987; Adami et al, 1994). There are 2 major different histo-pathological types, seminomas and non-seminomas, the latter form having an age-specific incidence peak 10 years earlier (25–29 years of age) than the seminomas (35–39 years of age) (Forman and Moller, 1994). The incidence rates of testicular cancer have increased during recent years in many countries including Great Britain (Coleman et al, 1993; Adami et al, 1994; Forman and Moller, 1994; Devesa et al, 1995), although there is some evidence of a leveling off of the incidence after 1990 (Pharris-Ciurej et al, 1999). Studies in several countries have shown that the increasing incidence of testicular cancer is strongly associated with birth cohort (Hoff Wanderas et al, 1995; Swerdlow et al, 1998; Liu et al, 1999; McKiernan et al, 1999).

There is a geographical variation in the incidence rates worldwide. The highest incidence rates have been noted in Denmark, Norway and Switzerland, whereas the lowest rates are seen in Eastern Europe and Asia (Akre, 1999). An almost 10-fold geographic variation within the Baltic sea countries has been observed (Adami et al, 1994; Ekbom and Akre, 1998).

There is concern that the increase in testicular cancer may be linked to environmental exposure to chemicals, in particular to so-called endocrine disrupting chemicals (such as dioxin, organochlorine, pesticides and PCBs), and evidence suggests that causal factors operate early in life, perhaps even in utero (Sharpe and Shaakebaek, 1993; Sonnenschein and Soto, 1998). Human exposure to chemicals in the environment is unlikely to be evenly distributed geographically, and if environmental chemicals have adverse effects on male reproductive health, they may thus give rise to geographical clustering of disease.

The aim of this study was to assess temporal trends and analyse spatial variations in testicular cancer at a small area level across Great Britain.

*Received 16 October 2000*
*Revised 28 December 2000*
*Accepted 2 January 2001*

*Correspondence to:* L Jarup

### MATERIALS AND METHODS

Registrations and mortality for testicular cancer (ICD-9 code 186), ages 20–49, for the whole of England, Wales and Scotland were extracted from the national post-coded data set held by the UK Small Area Health Statistics Unit (SAHSU). Cancer registration data were included between 1974 and 1991 and mortality data between 1981 and 1997. Ward-level denominator populations from the 1981 census were used for 1974–1981, 1991 census data were used from 1991–1997, and linear interpolation of the 1981 and 1991 census counts was used to estimate ward-level populations for 1982–1990. All small-area population estimates were then re-scaled to sum to the district-level Registrar General's population estimates for each year (Arnold, 1999).

Spatial variation in incidence was analysed across electoral wards, 1975 to 1991. All cases in the selected data set had valid postcodes. Data for 1974 were excluded because postcodes in Scotland were not established in cancer registration until 1975. For one ward in England, 42 cases of testicular cancer were recorded. Further investigation showed that these cases were assigned the geographical co-ordinates of a local military hospital and, therefore, this ward was excluded. No other potentially spurious cluster was detected. In total, 10 530 wards in Great Britain were included.

The Carstairs' index (Carstairs and Morris, 1991) was used as an index of relative deprivation at the electoral ward level. This index is a combination of 4 socio-economic indicators from the census – the percentage of people with no car, in overcrowded housing, with the head of household in social class IV or V, and the percentage of men unemployed. Population density was used as a measure of urbanization. Wards were grouped into quintiles of Carstairs' and population density score.

### Statistical methods

Temporal trends in incidence and mortality across Great Britain were examined using 3-year moving averages. For the spatial analysis, Poisson regression, allowing for over-dispersion, was used to analyse the ward-level relationship between age-standardized risk of incident cancer and deprivation, population density and regional cancer registry. To investigate for registry effects, the national average was chosen as the reference in the Poisson models. Statistical significance was assessed using likelihood ratio tests.

Ward-specific expected numbers of cases were then re-calculated to standardize for regional cancer registry and deprivation as well as age using national rates. Heterogeneity (excess variation) of disease rates was examined using the test described by Potthoff and Whittinghill (Potthoff and Whittinghill, 1966). This tests the hypothesis of homogeneity of risk against the alternative that the relative risks are drawn from a gamma distribution. Further analysis used Bayesian disease-mapping techniques to stabilize risk estimates based on small numbers at ward level (Mollie, 1996; Wakefield et al, 2000). Bayesian hierarchical modelling was first used to produce globally smoothed estimates of risk for all wards in Great Britain (Mollie, 1996). This analysis takes no account of possible spatially structured variation in risk of testicular cancer, so we then extended the hierarchical model to produce estimates of both spatially neutral variation (leading to global smoothing of risk as before) and spatially structured variation (leading to local smoothing of risk) (Besag et al, 1991). These analyses are highly computer intensive. For this reason and for clarity of presentation, this latter analysis was performed in 2 sub-regions, covered by the North West Thames registry (in the London area) and the Yorkshire registry, and were chosen because they represent a diverse mix of urban, suburban and rural populations.

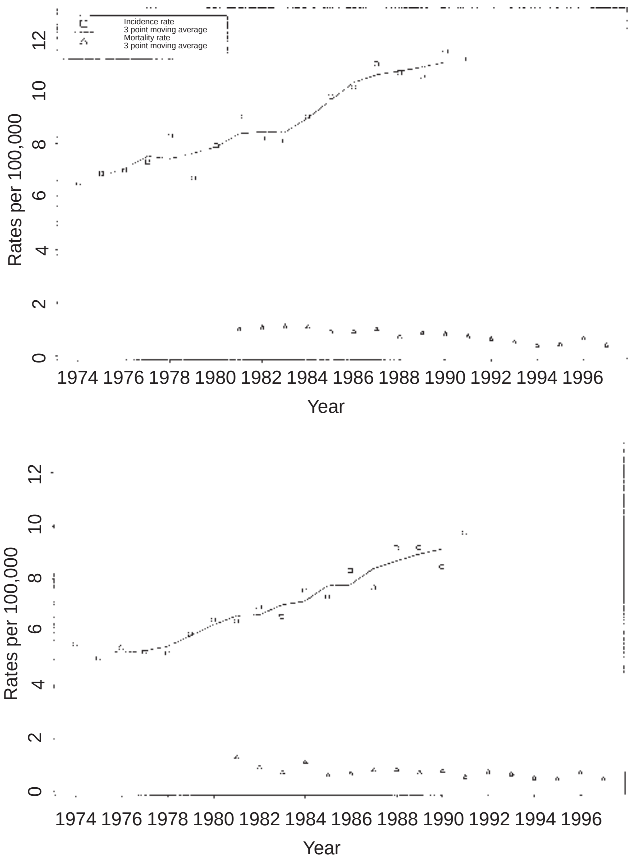

**Figure 1** Annual incidence and mortality rates for testicular cancer in Great Britain, 1974–1997, age 20–34 years (top) and age 35–49 years (bottom)

### RESULTS

The number of testicular cancer cases registered in Great Britain increased from 662 in 1974 to 1290 in 1991. Figure 1 shows the annual rates and 3-year moving averages for testicular cancer incidence (1974–1991) and mortality (1981–1997). The incidence rose from 6.5 to 11 per 100 000 in the younger age group (20–34, Figure 1), and from 5.6 to 9.7 per 100 000 in the older age group (35–49, Figure 1), i.e. almost a doubling of the incidence between 1974 and 1991. In contrast, there was over 50% reduction in mortality from testicular cancer in both age groups between 1981 (1.0 per 100 000 in the younger and 1.3 per 100 000 in the older age group) and 1996 (0.4 per 100 000 in both age groups).

Table 1 shows the relative risks for testicular cancer incidence by regional cancer registry adjusted for age and deprivation (Carstairs' quintiles). This shows significant variation by cancer registry, with relative risk estimates ranging from 0.8 (95% CI: 0.7–0.8) in NE Thames to 1.3 (95% CI: 1.3–1.4) in Scotland. There was also a significant association with deprivation: the estimated relative risk of testicular cancer was highest (RR = 1.3, 95% CI 1.2–1.4) in the most affluent compared with the most deprived quintile, after adjusting for age and cancer registry. Population density did not significantly improve the fit of this model, although it was significantly ($P < 0.05$) associated with testicular cancer incidence when deprivation was not included.

Table 2 summarizes the numbers of cases and the geographical variation in the relative risk estimates across wards in Great Britain. There was significant heterogeneity (Potthoff and Whittinghill test, $P < 0.0001$) in risk across wards, i.e. the variability in the observed relative risks was greater than that expected by chance if the rate ratios were the same for all areas in the study. However, as expected, the smoothed relative risks showed considerably less variation between the 5th and 95th percentiles (from 0.93 to 1.10) than the unsmoothed risk estimates (0 to 1.53). When maps of raw SMRs are plotted they are often dominated by sampling variability, and the SMRs were therefore 'smoothed' in an attempt to eliminate the spurious noise. The smoothing may be carried out globally or locally: in the former the rates are assumed to arise as an independent sample from a probability distribution, while in the latter spatial dependence is assumed. Global smoothing corresponds to the belief that all rates are similar while local smoothing to a belief that rates from areas that are geographically close are similar.

Tables 3 and 4 summarize the smoothed relative risks for the North West Thames and Yorkshire subsets of the data, allowing for spatially neutral (globally smoothed) and/or spatially structured (locally smoothed) variation in risk.

Model comparison was based on the deviance information criterion (DIC) (Spiegelhalter et al, 1998). This is given by a term for model fit (assessed via the model deviance) plus a penalty term for

© 2001 Cancer Research Campaign model complexity, with lower values indicating better fit. The Bayesian approach allows the posterior probability of any area's relative risks exceeding a threshold to be calculated. In NW Thames, 20 wards showed an estimated relative risk greater than unity with at least 90% probability, with 2 of these wards having at least 95% probability of the relative risk exceeding 1.0. There were no wards in Yorkshire for which the estimated relative risk exceeded 1.0 with at least 90% probability. Figure 2 shows the age- and deprivation-adjusted unsmoothed and smoothed relative risks for electoral wards for the Yorkshire and NW Thames subsets (using smoothed estimates from the best-fitting model for each region).

**Table 2** Spatial variation in relative risks (RR) of testicular cancer adjusted for age, relative deprivation[a] and cancer registry across 10 530 electoral wards in Great Britain (1975–1991), using hierarchical Bayesian methods for the smoothed estimates

| | Observed | Expected | Unsmoothed RR | Smoothed RR |
|---|---|---|---|---|
| Minimum | 0 | 0.01 | 0 | 0.79 |
| 5th percentile | 0 | 0.59 | 0 | 0.93 |
| Median | 1 | 1.15 | 0.78 | 0.99 |
| 95th percentile | 2 | 1.98 | 1.53 | 1.10 |
| Maximum | 35 | 9.14 | 29.20 | 2.81 |
| Mean | 1.45 | 1.45 | 1.01 | 1.00 |

[a] Using Carstairs' scores.

### DISCUSSION

Recent concerns about possible environmental causes of testicular cancer have highlighted the need for a current appraisal of its epidemiology. This study is the first to examine both temporal trends and geographical variation of testicular cancer at both regional and small-area scales.

**Table 1** Age-adjusted relative risks for testicular cancer (1975–1991) by cancer registry and deprivation, expressed as quintiles of the Carstairs' deprivation index (CQ)[a, b]

| Cancer registry | RR | 95% CI |
|---|---|---|
| East Anglia | 1.13 | 1.05–1.22 |
| Mersey | 1.00 | 0.92–1.08 |
| Northern | 0.90 | 0.84–0.96 |
| North Western | 0.99 | 0.93–1.06 |
| Oxford | 1.16 | 1.09–1.24 |
| South Western | 1.11 | 1.04–1.18 |
| Trent | 0.96 | 0.91–1.02 |
| Thames: | | |
| NE Thames | 0.79 | 0.73–0.84 |
| NW Thames | 0.88 | 0.83–0.94 |
| South Thames | 0.92 | 0.88–0.97 |
| Yorkshire | 1.01 | 0.95–1.07 |
| Wessex | 1.13 | 1.06–1.21 |
| West Midlands | 0.92 | 0.87–0.97 |
| Scotland | 1.32 | 1.25–1.38 |
| Wales | 0.90 | 0.83–0.97 |
| CQ5 (most deprived) | 1.00 | |
| CQ4 | 1.14 | 1.08–1.19 |
| CQ3 | 1.15 | 1.09–1.21 |
| CQ2 | 1.23 | 1.17–1.30 |
| CQ1 (most affluent) | 1.31 | 1.24–1.38 |

[a] CQ5 was used as a reference. The risk estimates for cancer registry and deprivation in the table were mutually adjusted. [b]Dispersion parameter = 1.10 (or 10% over dispersion).

### Time trends

The present study showed an increasing temporal trend in testicular cancer incidence, with an approximate doubling of the rates in both age groups from 5–6 per 100 000 in 1974 to 10–11 per 100 000 in 1991. This is consistent with reports from several other countries, showing that the incidence of testicular cancer is rising similarly in most Western populations (Adami et al, 1994). In the west of Scotland, the number of germ cell tumours registered more than doubled between 1960 and 1990 (Hatton et al, 1995). A study from Norway reported that the age-standardized incidence for testis cancer increased from 2.7 per 100 000 in 1955 to 8.5 per 100 000 in 1992 (Hoff Wanderas et al, 1995). In contrast, testicular cancer mortality showed a sharp decline, in accordance with the findings from other countries (Devesa et al, 1987; Forman and Moller, 1994). The reason for the increasing incidence is still unclear, although environmental chemicals, in particular xenoestrogens (Sharpe and Skakkebaek, 1993), as well as infections (Swerdlow et al, 1987) have been suggested, while the decreasing mortality must largely reflect advances in treatment, particularly the introduction of chemotherapy including cis-platinum (Osterlind, 1986; Chu et al, 1991).

**Table 3** Spatial variation in unsmoothed and smoothed relative risks[a] (RR) of testicular cancer, adjusted for age and deprivation, across 532 electoral wards in the North West Thames cancer registry, 1975–1991

| | Observed | Expected | Unsmoothed RR | Smoothed RR | | |
|---|---|---|---|---|---|---|
| | | | | Spatially neutral variation | Spatially structured variation | Spatially neutral & structured variation |
| Minimum | 0 | 0.33 | 0 | 0.92 | 0.91 | 0.89 |
| 5th percentile | 0 | 0.56 | 0 | 0.97 | 0.92 | 0.90 |
| Median | 1 | 1.69 | 0.89 | 1.00 | 0.99 | 0.99 |
| 95th percentile | 5 | 3.38 | 2.61 | 1.04 | 1.15 | 1.16 |
| Maximum | 8 | 4.86 | 5.83 | 1.14 | 1.27 | 1.31 |
| Mean | 1.79 | 1.79 | 1.03 | 1.00 | 1.00 | 1.00 |
| Model fit, DIC[b] | – | – | – | 601.6 | 594.6 | 593.8 |

[a]Using hierarchical Bayes' methods. [b]Deviance Information Criterion.

 © 2001 Cancer Research Campaign

**Table 4** Spatial variation in unsmoothed and smoothed relative risks[a] (RR) of testicular cancer, adjusted for age and deprivation, across 525 wards in Yorkshire cancer registry, 1975–1991

| | Observed | Expected | Unsmoothed RR | Smoothed RR | | |
|---|---|---|---|---|---|---|
| | | | | Spatially neutral variation | Spatially structured variation | Spatially neutral & structured variation |
| Minimum | 0 | 0.20 | 0 | 0.95 | 0.92 | 0.90 |
| 5th percentile | 0 | 0.36 | 0 | 0.98 | 0.92 | 0.91 |
| Median | 1 | 1.10 | 0.87 | 1.00 | 1.01 | 1.00 |
| 95th percentile | 6.6 | 5.22 | 2.87 | 1.03 | 1.02 | 1.04 |
| Maximum | 11 | 8.09 | 6.30 | 1.09 | 1.02 | 1.11 |
| Mean | 1.85 | 1.85 | 0.99 | 1.00 | 1.00 | 0.99 |
| Model fit, DIC[b] | – | – | – | 565.3 | 566.0 | 567.2 |

[a]Using hierarchical Bayes' methods. [b]Deviance Information Criterion.

### Geographical variation

Overall, we found statistically significant geographical differences in the risk of testicular cancer at regional level, although there was no particular spatial pattern to the between-registry variation. In addition there was no clear evidence of local spatial variation at small area level.

We standardized the expected counts for this analysis by registry to account for possible differences between cancer registries in case ascertainment (Swerdlow, 1986; Best and Wakefield, 1999). Within registries there may be local variations in ascertainment that we were not able to account for, and which would add to the observed variability at small area scale.

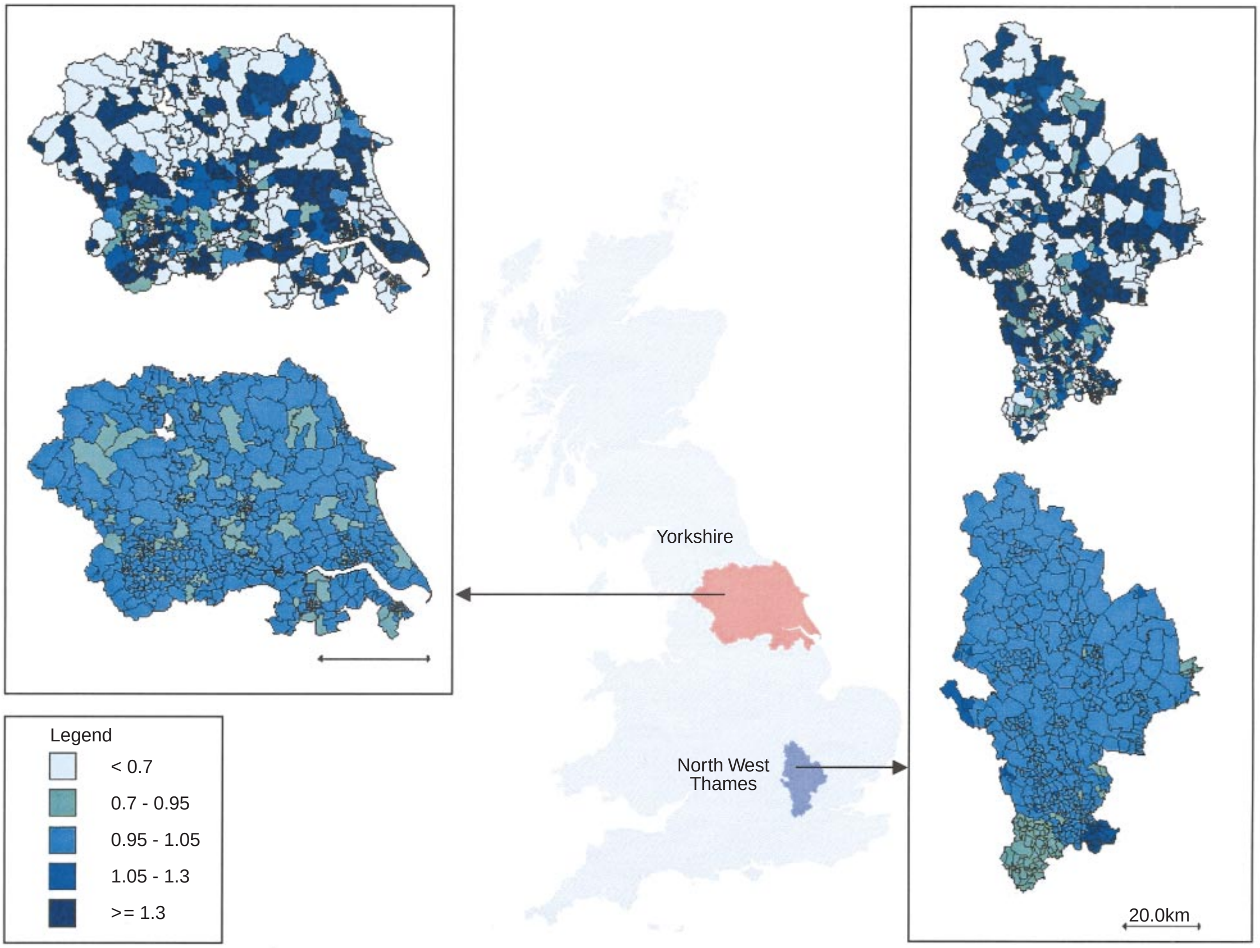

**Figure 2** Age- and deprivation-adjusted relative risks of testicular cancer, 1975–1991, across electoral wards in Yorkshire cancer registry, unsmoothed risks (top left) and after smoothing (bottom left), and in the North West Thames cancer registry, unsmoothed risks (top right) and after smoothing (bottom right)

© 2001 Cancer Research Campaign 

Further difficulties in interpreting geographical variation include the long latency periods of this cancer and the effects of migration. In particular, in-utero exposure is likely to be important for testicular cancer risk, and it has been shown that the geographic pattern of incidence is stronger for residence at birth than for residence at the time of diagnosis (Moller, 1997). The residence of mothers during pregnancy may be quite different from the residence of young men at the time of diagnoses. Thus, latency times and migration patterns are likely to reduce the size of estimated spatial differences in risk.

Previous studies have reported geographical variations of testicular cancer incidence. A collaborative study between 9 population-based cancer registries in countries around the Baltic Sea found a substantial variation in the age-standardized incidence of testicular cancer (Adami et al, 1994). There was a 10-fold difference between the different countries ranging from 0.8 per 100 000 person-years in Lithuania to 7.6 per 100 000 person-years in Denmark. However, the authors note that the marked heterogeneity in incidence between the countries contrasts with a marked homogeneity within each country as demonstrated from the Nordic countries (Jensen et al, 1988). The difference between the Nordic and the Baltic countries suggests that the observed heterogeneity between countries may reflect differences in registration procedures, rather than true differences. Another possible explanation would be variation in socio-economic conditions between the Nordic and the Eastern European countries. However, differences in registration procedures or socio-economic status cannot readily explain the 6-fold variation between the Nordic countries, but so far no plausible explanation for this variation has been given. The authors also suggest that early exposure to environmental agents varying between countries may be responsible for the differences (Adami et al, 1994). So far, however, it has not been possible to pinpoint any environmental chemical with spatially different concentration levels between countries. For example, there were no differences in the DDT-metabolite p,p′ DDE (an androgen receptor antagonist) in breast milk, over time between the 4 Nordic countries (Ekbom et al, 1996).

In a study of geographical variation at county level in England and Wales, testicular cancer risk tended to be higher in the south of England amongst the 0–49 year age group, but there was no consistent pattern (Swerdlow and dos Santos Silva, 1993). We found the lowest risks in North East Thames (RR = 0.79, 95% CI: 0.73–0.84) and the highest risks in Scotland (RR = 1.32, 95% CI: 1.25–1.38), but no consistent north–south gradient. The lower risk in North East Thames may, at least partly, reflect relative under-ascertainment, which was a particular problem with this registry prior to 1986 (Best and Wakefield, 1999; Bullard et al, 2000).

We found significantly higher testicular cancer risk with decreasing relative deprivation at the small-area level, in contrast to previous studies at individual level, which have found no evidence of any social class effect (UK Testicular Cancer Study Group, 1994).

In our study, population density was associated with testicular cancer incidence but was no longer significant with adjustment for deprivation, suggesting that any urban–rural difference was reflecting differences in socio-economic status at a small-area scale. Similarly, a recent Dutch study found no urban–rural differences, although there was a suggestion of geographical clustering in a rural area in the north of the Netherlands (Sonneveld et al, 1999).

Our analysis within the Yorkshire and North West Thames registries did not suggest any marked geographical clustering of testicular cancer incidence at the level of electoral wards. While it is difficult to judge whether the findings for these 2 registries are representative, the 5th to 95th percentile range of smoothed risk estimates across each region in the Bayesian analysis was similar to that for Britain as a whole.

Interpretation of geographical variations is complicated by a number of factors. These include variations in data quality between and within regions, long latency periods and migration effects, which are likely to reduce the ability to detect any true variation. Nonetheless, the absence of any marked geographical variability at small area scale argues against a geographically varying environmental factor operating strongly in the aetiology of testicular cancer. However, this does not preclude an important role of a ubiquitous (environmental) exposure, which may have strong variability at the national level.

### ACKNOWLEDGEMENTS

The Small Area Health Statistics Unit is funded by a grant from the Department of Health, Department of the Environment, Transport and The Regions, Environment Agency Health and Safety Executive, Scottish Executive, National Assembly for Wales and Northern Ireland Department of Health Social Services and Public Safety. We thank the Office for National Statistics for provision of and permission to use the mortality and cancer incidence data. The views expressed in this publication are those of the authors and not necessarily of the funding departments. We also wish to express our thanks to Ms Cathy Wallace who performed the initial heterogeneity analysis.

### REFERENCES

Adami HO, Bergstrom R, Mohner M, Zatonski W, Storm H, Ekbom A, Tretli S, Teppo L, Ziegler H and Rahu M (1994) Testicular cancer in nine northern European countries. *Int J Cancer* **59**: 33–38

Akre O (1999) *Etiological insights into the testicular cancer epidemic*. Stockholm: Karolinska institutet

Arnold RA (1999) Small Area Health Statistics Unit procedures for estimating populations in small areas. In: Arnold R, Elliott P, Wakefield J, Quinn M. (eds) *Population counts in small areas: implications for studies of environment and health*, pp 10–24. HMSO: London

Besag J, York J and Mollie A (1991) Bayesian image restoration, with application in spatial statistics with discussion). *Annals of the Institute of Statistical Mathematics* **43**: 1–59

Best N and Wakefield J (1999) Accounting for inaccuracies in population counts and case registration in cancer mapping studies. *Journal of the Royal Statistical Society A* **162**: 363–382

Bullard J, Coleman MP, Robinson D, Lutz JM, Bell J and Peto J (2000) Completeness of cancer registration: a new method for routine use. *Br J Cancer* **82**: 1111–1116

Carstairs V and Morris R (1991) *Socio-economic status and health in Scotland*. Aberdeen: Aberdeen University Press

Chu KC, Kramer BS and Smart CR (1991) Analysis of the role of cancer prevention and control measures in reducing cancer mortality. *J Natl Cancer Inst* **83**: 1636–1643

Coleman MP, Esteve J, Damiecki P, Arslan A and Renard H (1993) *Trends in Cancer Incidence and Mortality*. Lyon: International Agency for Research on Cancer

Devesa SS, Silverman DT, Young Jr JL, Pollack ES, Brown CC, Horm JW, Percy CL, Myers MH, McKay FW and Fraumeni Jr JF (1987) Cancer incidence and mortality trends among whites in the United States, 1947–84. *J Natl Cancer Inst* **79**: 701–770

Devesa SS, Blot WJ, Stone BJ, Miller BA, Tarone RE and Fraumeni Jr JF (1995) Recent cancer trends in the United States [see comments]. *J Natl Cancer Inst* **87**: 175–182

Ekbom A, Wicklund-Glynn A and Adami HO (1996) DDT and testicular cancer [letter; comment]. *Lancet* **347**: 553–554

Ekbom A and Akre O (1998) Increasing incidence of testicular cancer – Birth cohort effects. APMIS **106**: 225–231

 *© 2001 Cancer Research Campaign*

Forman D and Moller H (1994) Testicular cancer. [Review] [39 refs]. *Cancer Surv* 19–20: 323–341

Hatton MQ, Paul J, Harding M, MacFarlane G, Robertson AG and Kaye SB (1995) Changes in the incidence and mortality of testicular cancer in Scotland with particular reference to the outcome of older patients treated for non-seminomatous germ cell tumours. *Eur J Cancer* **31A**: 1487–1491

Hoff Wanderas E, Tretli S and Fossa SD (1995) Trends in incidence of testicular cancer in Norway 1955–1992. *Eur J Cancer* **31A**: 2044–2048

Jensen O, Carstensen B, Glattre E, Malker B, Pukkala E and Tulinius H (1988) *Atlas of cancer incidence in the Nordic countries*. Helsinki: Nordic Cancer Union.

Liu S, Wen SW, Mao Y, Mery L and Rouleau J (1999) Birth cohort effects underlying the increasing testicular cancer incidence in Canada. Canadian Journal of Public Health Revue Canadienne de Sante Publique/Can J Public Health **90**: 176–180

McKiernan JM, Goluboff ET, Liberson GL, Golden R and Fisch H (1999) Rising risk of testicular cancer by birth cohort in the United States from 1973 to 1995. *J Urol* **162**: 361–363

Moller H (1997) Work in agriculture, childhood residence, nitrate exposure, and testicular cancer risk: a case-control study in Denmark. *Cancer Epidemiol Biomarkers Prev* **6**: 141–144

Mollie A (1996) Bayesian mapping of disease. In: Gilks WR, Richardson S, Spiegelhalter DJ (eds) *Markov Chain Monte Carlo in Practice*, pp 359–379. Chapman and Hall: London

Osterlind A (1986) Diverging trends in incidence and mortality of testicular cancer in Denmark, 1943–1982. *Br J Cancer* **53**: 501–505

Pharris-Ciurej ND, Cook LS and Weiss NS (1999) Incidence of testicular cancer in the United States: has the epidemic begun to abate? *Am J Epidemiol* **150**: 45–46

Potthoff R and Whittinghill M (1966) Testing for Homogeneity. I. The binimial and multinomial distributions and II. The poisson distribution. *Biometrika* **53**: 167–190

Senturia YD (1987) The epidemiology of testicular cancer. [Review] [29 refs]. *Br J Urol* **60**: 285–291

Sharpe RM and Skakkebaek NE (1993) Are oestrogens involved in falling sperm counts and disorders of the male reproductive tract? [see comments]. *Lancet* **341**: 1392–1395

Sonnenschein C and Soto AM (1998) An updated review of environmental estrogen and androgen mimics and antagonists. [Review] [74 refs]. *J Steroid Biochem Mol Biol* **65**: 143–150

Sonneveld DJ, Schaapveld M, Sleijfer DT, Meerman GJ, van der Graaf WT, Sijmons RH, Koops HS and Hoekstra HJ (1999) Geographic clustering of testicular cancer incidence in the northern part of The Netherlands. *Br J Cancer* **81**: 1262–1267

Spiegelhalter DJ, Best NG and Carlin BP (1998) *Technical report*. (http://www.mrc-bsu.cam.ac.uk/Publications/preslid.shtml). Cambridge: MRC Biostatistics Unit

Swerdlow AJ (1986) Cancer registration in England and Wales: some aspects relevant to interpretation of the data. *Journal of the Royal Statistical Society A* **149**: 146–160

Swerdlow A and dos Santos Silva I (1993) *Atlas of cancer incidence in England and Wales 1968–85*. Oxford: Oxford University Press

Swerdlow AJ, Huttly SR and Smith PG (1987) Testicular cancer and antecedent diseases. *Br J Cancer* **55**: 97–103

Swerdlow AJ, dos Santos Silva I, Reid A, Qiao Z, Brewster DH and Arrundale J (1998) Trends in cancer incidence and mortality in Scotland: description and possible explanations. *Br J Cancer* 77 Suppl **3**: 1–54

UK Testicular Cancer Study Group (1994) Social, behavioural and medical factors in the aetiology of testicular cancer: results from the UK study. *Br J Cancer* **70**: 513–520

Wakefield JC, Best NG and Waller L (2000) Bayesian approaches to disease mapping. In: Elliott P, Wakefield JC, Best NG, Briggs D (eds) *Spatial Epidemiology: Methods and Applications*. pp 104–127 Oxford University Press: Oxford

© 2001 Cancer Research Campaign
